# Supplementary material for: Flow Cytometric Analysis of Oxidative Stress in Escherichia coli B Strains Deficient in Genes of the Antioxidant Defence
Source: Int J Mol Sci. 2022 Jun 10;23(12):6537. doi: 10.3390/ijms23126537 (PMC9223410; doi:10.3390/ijms23126537)
Supplement: Supplementary file 1 [file ijms-23-06537-s001.zip › ijms-1722639-supplementary.pdf]

Supplementary Figure 1

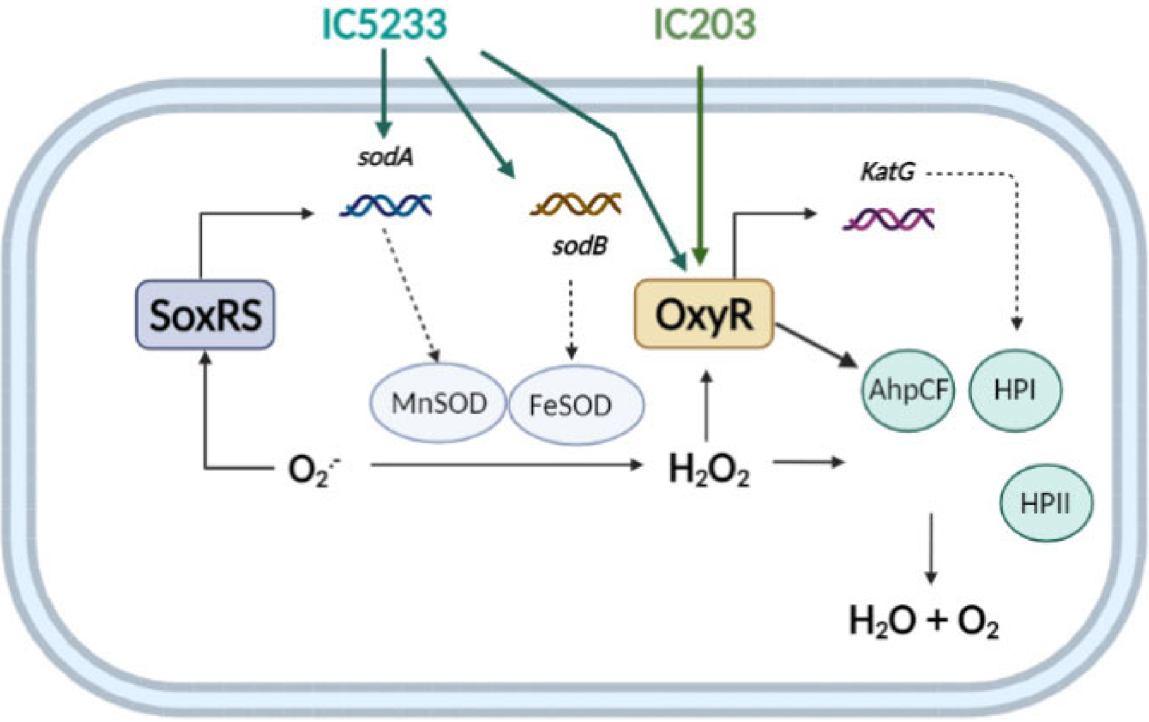

Figure S1. Scheme of the genetic modifications induced on wildtype strain IC188 to generate the strains IC203 (deficient in oxyR) and IC5233 (deficient in oxyR, sodA and sodB).

Supplementary Figure 2

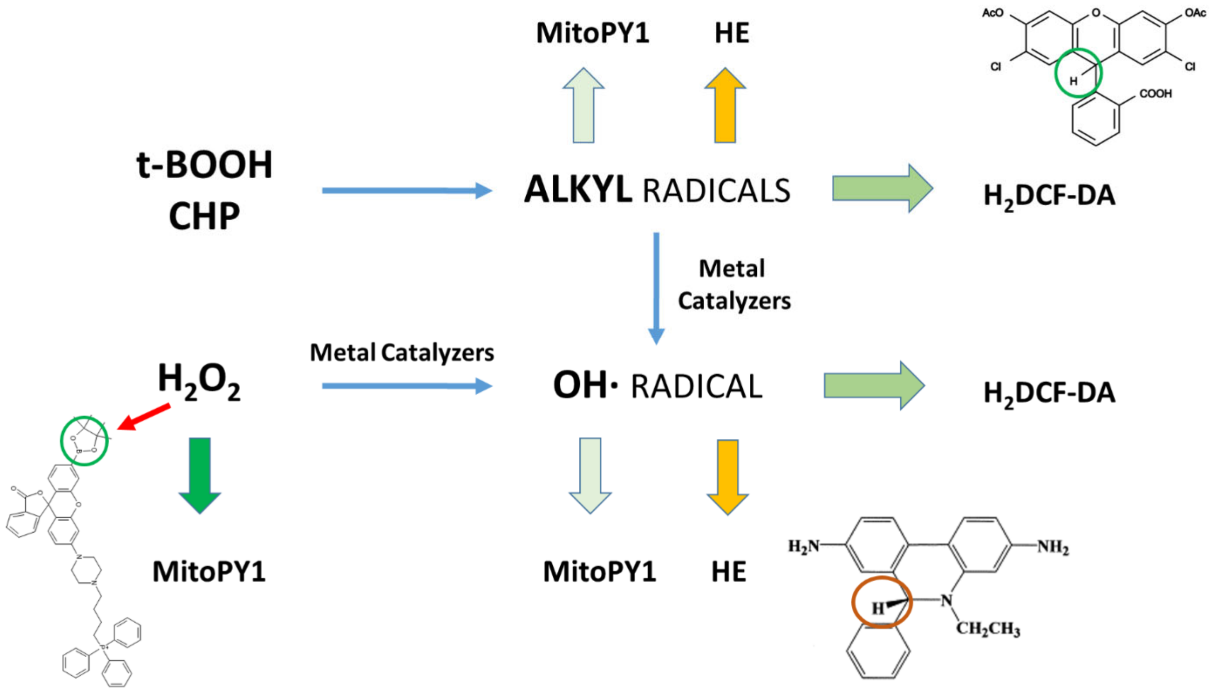

Figure S2. Scheme of the oxidative processes involved in the interaction among the fluorescent probes and peroxides used in this study. MitoPY1 undergoes chemospecific reaction with H2O2 at the boronate moiety (circle) to emit green fluorescence. H2-DCF and HE undergo ROS-mediated loss of a H atom (circles) to yield green- and orange emitting fluorochromes (circles).
